# Biodegradable magnesium Herbert screw – image quality and artifacts with radiography, CT and MRI

**DOI:** 10.1186/s12880-017-0187-7

**Published:** 2017-02-14

**Authors:** Lena Sonnow, Sören Könneker, Peter M. Vogt, Frank Wacker, Christian von Falck

**Affiliations:** 1 Department of Diagnostic and Interventional Radiology, Hannover, 30625 Germany; 2 Department of Plastic, Hand and Reconstructive Surgery, Hannover Medical School, Carl-Neuberg-Str. 1, 30625 Hannover, Germany

## Abstract

**Background:**

Magnesium alloys have recently been rediscovered as biodegradable implants in musculoskeletal surgery. This study is an ex-vivo trial to evaluate the imaging characteristics of magnesium implants in different imaging modalities as compared to conventional metallic implants.

**Methods:**

A CE-approved magnesium Herbert screw (MAGNEZIX®) and a titanium screw of the same dimensions (3.2x20 mm) were imaged using different modalities: digital radiography (DX), multidetector computed tomography (MDCT), high resolution flat panel CT (FPCT) and magnetic resonance imaging (MRI). The screws were scanned in vitro and after implantation in a fresh chicken tibia in order to simulate surrounding bone and soft tissue. The images were quantitatively evaluated with respect to the overall image quality and the extent and intensity of artifacts.

**Results:**

In all modalities, the artifacts generated by the magnesium screw had a lesser extent and were less severe as compared to the titanium screw (mean difference of artifact size of solo scanned screws in DX: 0.7 mm, MDCT: 6.2 mm, FPCT: 5.9 mm and MRI: 4.73 mm; p < 0.05). In MDCT and FPCT multiplanar reformations and 3D reconstructions were superior as compared with the titanium screw and the metal-bone interface after implanting the screws in chicken cadavers was more clearly depicted. While the artifacts of the titanium screw could be effectively reduced using metal-artifact reduction sequences in MRI (WARP, mean reduction of 2.5 mm, p < 0.05), there was no significant difference for the magnesium screw.

**Conclusions:**

Magnesium implants generate significantly less artifacts in common imaging modalities (DX, MDCT, FPCT and MRI) as compared with conventional titanium implants and therefore may facilitate post-operative follow-up.

**Electronic supplementary material:**

The online version of this article (doi:10.1186/s12880-017-0187-7) contains supplementary material, which is available to authorized users.

## Background

After the discovery of elemental magnesium (Mg) by Sir Humphrey Davy in 1808 Mg-implants were initially used in musculoskeletal surgery in the first half of the twentieth century [1]. In 1900, the Austrian-German physician Erwin Payr proposed possible Mg-implants such as nails, wires, pegs, sheets and plates for internal stabilization of bone fractures [2]. There are several case reports of in vivo magnesium application in patients who had suffered from supracondylar fractures, femoral fractures or pseudarthrosis and who could successfully be treated with Mg-implants [3].

Nevertheless, the rapid degradation of the implants causing a distinct hydrogen gas formation was a common and major complication. The inability to control the rate of degradation in vivo led to the abandonment of magnesium and its replacement by inert and corrosion-resistant materials such as stainless steel for orthopedic implants [3, 4]. However, in the last few years, magnesium based alloys were rediscovered as biodegradable implants and have attracted increasing interest acting as “smart” implants belonging to the “third generation” of orthopedic biomaterials [5, 6]. Magnesium alloys not only possess the ability to degrade but also to stimulate cellular responses at the molecular level. Animal experiments demonstrated not only a complete degradation but moreover an increase in bone mass and mineral apposition rate around the implant, suggesting magnesium having osteoinductive properties [7, 8]. Recent developments in alloying adjustment, surface treatments and coating technologies provided an improvement of mechanical properties and a significant reduction of the magnesium corrosion rate. Thus, the combination of good biomechanics, the ability to control degradation as well as biocompatibility and bioactivity turn magnesium into a promising biomaterial in orthopedics. Being a stable implant that degrades in vivo, magnesium may improve fracture healing and eliminate the need for a second operation for implant removal.

In 2013 the MAGNEZIX ® compression screw emerged as the first commercially available, CE-approved Mg-based orthopedic product. A prospective clinical study demonstrated that the degradable Mg-based screws are equivalent to titanium screws for the treatment of mild hallux valgus deformities [9]. Another possible field of application of the Mg-based compression screw is the treatment of scaphoid fractures. As an implant with osteoinductive properties it could facilitate bony healing and prevent the common complication of nonunion. In postoperative care, especially the extent of bony consolidation and the first signs of pseudarthrosis require a very precise imaging diagnostic. The image quality, however, often is impeded by artifacts of the metallic implant and therefore complicates evaluation of the surrounding tissue. A major advantage of Mg-based biomaterials is their property to cause only little artifacts in the common imaging modalities. However, there is very limited experience with imaging of Mg-based orthopedic materials.

The objective of this study was to explore the imaging properties of a magnesium based Herbert compression screw and to gain knowledge about the optimal imaging parameters in order to achieve maximal artifact reduction and image quality with in vivo applicable imaging modalities.

## Methods

A cannulated compression Herbert screw with the size of 3.2 x 20 mm consisting of > 90 wt.% magnesium (MAGNEZIX® MgYREZr alloy, Syntellix AG, Hannover, Germany) and a conventional titanium Herbert screw (KLS, Martin) were obtained. In digital flat panel radiography (DX), multi-detector computed tomography (MDCT) and flat-panel computed tomography (FPCT) the screws were positioned on the scanning table without further preparations. For magnetic resonance imaging, the specimens were placed in ultrasound gel to ensure sufficient water signal. Following the assessment of artifacts of the solely scanned screws, they were implanted in chicken bone for more physiological conditions.

### Preparation of specimens

Two chicken lower leg cadavers were acquired for the study as the dimensions best align with a human wrist. A midline longitudinal incision of about 3 cm was performed at the proximal end of the leg and soft tissue was dissected to expose the tibial corticalis. Either the magnesium or the titanium Herbert screw of the same dimensions (3.2 x 20 mm, cannulated) were inserted in a predrilled hole in each tibia perpendicularly to the tibial axis and fixated bicortically. The soft tissue was readapted and the position of the screw was approximately marked on the chicken skin. In addition a lesion of about 0.5 x 0.5 mm extent was artificially created in the cartilage of the tibial plateau. Each lower leg was sealed in a plastic bag and transmitted to scanning.

### DX

Conventional radiography in two planes was acquired with parameters adjusted to the small size of the chicken specimens (tube voltage 50 kV, tube current 3 mAs, distance source to specimen 609 mm, distance source to detector 663 mm).

### MDCT

The specimens were positioned on the scanning table of a 16-slice MDCT (LightSpeed16, GE Healthcare, UK) in two different positions by aligning the central screw axis either with the x- or z-direction of the scanner. A routine wrist scanning protocol with the following parameters was used: tube voltage 120 kV, X-ray tube current 100 mA, slice thickness 0.625 mm, reconstruction increment 0.4 mm, FOV 9.6 cm, reconstruction: filtered back projection (‘bone plus’ kernel). The scans were replicated five times.

### FPCT

The FPCT scans were performed on a dedicated angiography unit (Artis Q, Siemens, Erlangen, Germany) with a C-arm-mounted FP detector (30 x 40 cm) operated in an ultra high-resolution 1x1 read out mode (‘DynaCT micro’). A 20 s protocol with a field of view of 22 cm was applied. Each specimen was scanned five times with the central screw axis aligning either with the x- or with the z-axis of the scanner.

### MRI

MRI scans were performed on a 1.5 T MRI Scanner (Siemens Avanto, Siemens Healthcare Erlangen, Germany) using an 8-channel wrist coil and on a 3 T MRI scanner (Siemens Skyra, Siemens Healthcare, Erlangen, Germany) using a 4-channel flex coil. The specimens were positioned in two orthogonal directions with the central screw axis aligned with the x- or z-direction of the scanner (main magnetic field parallel to the z-direction). Images were acquired perpendicularly (axial) to the screw axis. The choice of the sequences and parameters was made according to a standard wrist protocol which is applied in clinical routine: PD weighted (w) TSE fat saturated (FS) and T1w TSE sequences. In addition a metal artifact reduction were performed on the 3 T scanner: PDw TSE FS WARP. The scans were replicated five times. The scan parameters are given in detail in Table 1.

**Table 1:** MRI scanning parameters

Sequence	TR (ms)	TE (ms)	Flip Angle (°)	Band-width (Hz/pix)	Slice thickness (mm)	Slice spacing (mm)	FOV (mm)
Avanto (1.5 T):							
PDw TSE (FS)	2800	38	160	85	2.0	2.2	140
T1w TSE	571	13	180	130	2.0	2.2	140
Skyra (3 T):							
PDw TSE (FS)	2200	31	150	200	2.0	2.2	200
T1w TSE	869	11	150	257	2.0	2.2	200
PDw TSE WARP (FS)	2200	31	150	200	2.0	2.2	200

### Analysis of artifacts

All artifacts were assessed in an axial plane or reconstruction of the screw. In MDCT and FPCT radial artifacts with the largest extent were measured regardless of their orientation. In MRI artifacts aligning the y-axis (defined as the vertical axis of the scanner) were considered. As artifact appearance always was symmetrical, measurement was performed by creating a straight line through the outer boundaries of the artifacts and the central screw axis. The degree of artifact was defined as the length of the hypodense line emanating from the screw in MDCT/FPCT or as the diameter of the signal loss induced by the screw in MRI. When artifacts with various lengths were produced, the longest were measured. The same process was repeated in three different axial slices of the screw and the average value was obtained. For better orientation and comparability the artificial cartilage lesion served as reference, thus the slices in a similar position could be chosen. All images were viewed in a preset window level and width with the following values for window center and width (C/W): MDCT 300/1500 HU; FPCT 300/1000 HU; PDw TSE/T1w 650/1300; PDw WARP 400/800.

### Statistics

Measurements of artifact size were averaged across the slices and scans and compared for each modality and sequence with regard to the implanted screw. In order to assess whether there are significant differences in the size of artifacts generated between the two screw materials, an unpaired *t*-test was conducted. For evaluation of the artifact reduction sequences, a paired *t*-test was conducted for the WARP and non-WARP sequence for magnesium and titanium respectively. A p < 0.05 was considered statistically significant.

## Results

In all modalities, the artifacts generated by the magnesium screw had a lesser extent and were less severe as compared to the titanium screw. At first, artifact measurements were performed in the images of the solely scanned screws. In this setting, the mean difference of artifact size was: DX: 0.7 mm, MDCT: 6.2 mm, FPCT: 5.9 mm, MRI PDw TSE: 2.6 mm and MRI T1w: 4.5 mm with p < 0.005. Detailed results are given in Table 2.

**Table 2:** Artifact size of the solely scanned screws

Imaging modality	Mg: mean artifact size [mm]	Ti: mean artifact size [mm]	Mean difference in artifact size [mm]	T-value	*P*-value
DX	4.5 ± 0.3	5.2 ± 0.2	0.7 ± 0.2	4.1	<0.005
MDCT	5.7 ± 0.4	11.9 ± 0.5	6.2 ± 0.2	27.0	<0.001
FPCT	5.5 ± 0.2	11.4 ± 0.5	5.9 ± 0.2	31.8	<0.001
3 T MRI					
PDw TSE	5.2 ± 0.7	7.9 ± 0.5	2.6 ± 0.3	9.1	<0.001
T1w TSE	8.4 ± 0.7	12.9 ± 1.0	4.5 ± 0.4	10.6	<0.001
PDw TSE WARP	5.0 ± 0.7	6.2 ± 0.5	1.1 ± 0.3	3.6	<0.005

**Fig. 1:**
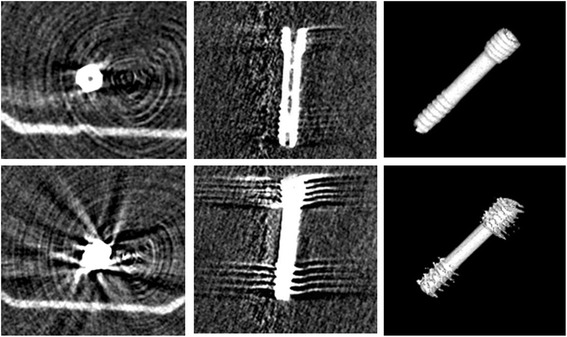
Mg-screw (top) and Ti-screw (bottom) in FPCT. The screw axis aligns to the z-axis of the scanner (0° position)

Figure 1 illustrates the artifacts in a FPCT scan of a magnesium and a titanium screw without any surrounding soft or bony tissue. Moreover, it demonstrates the superior image quality of a 3D reconstruction of the magnesium screw in comparison to titanium.

**Fig. 2:**
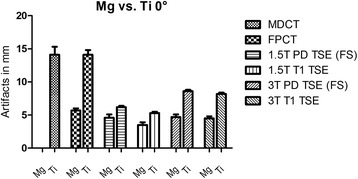
Artifacts (mm) in different modalities with a screw position at 0°

**Fig. 3:**
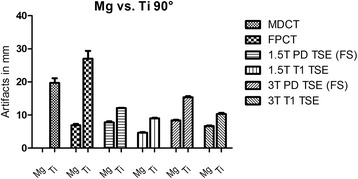
Artifacts (mm) in different modalities with a screw position at 90°

**Fig. 4:**
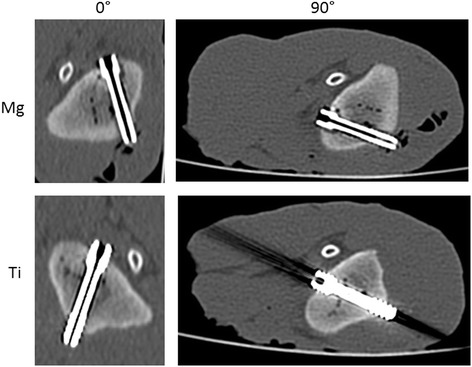
Mg-screw (top) and Ti-screw (bottom) in MDCT in the 0° and 90° degree positions

**Fig. 5:**
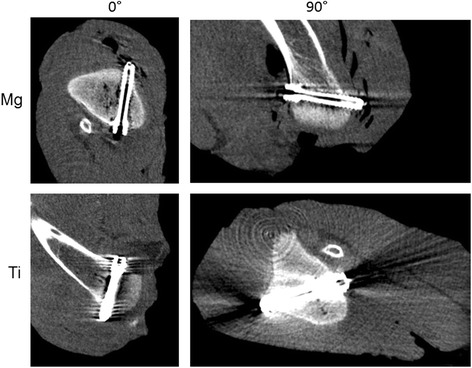
Mg-screw (top) and Ti-screw (bottom) in FPCT in the 0° and 90° degree positions

Similarly to the solo scans, the magnesium screw was clearly superior to titanium when implanted in chicken tibia with regard to artifact extent and signal distortion. In DX and MDCT the artifacts around the magnesium screw in chicken tibia were of such a small magnitude that they were innumerable and had to be considered zero. In addition, the screw position relatively to the longitudinal axis of the scanner (z-axis) contributed significantly to artifact occurrence. Therefore, artifact extent was compared separately for the parallel (0°) and perpendicular (90°) screw positions with a mean difference of artifact size between Mg and Ti for the 0°-position in MDCT: 14.1 mm, FPCT 8.5 mm, 1.5 T MRI PDw TSE (FS) 1.6 mm, 1.5 T MRI T1w 1.8 mm, 3 T MRI PDw TSE (FS) 3.9 mm and 3 T MRI T1w 3.7 mm. For the 90°-position the mean difference between Mg and Ti was in MDCT: 14.1 mm, FPCT 8.5 mm, 1.5 T MRI PDw TSE (FS) 1.6 mm, 1.5 T MRI T1w 1.8 mm, 3 T MRI PDw TSE (FS) 3.9 mm and 3 T MRI T1w 3.7 mm. In DX the mean artifact difference was 3.9 mm. With regard to the absolute artifact extent, the longitudinal orientation of the screw was significantly superior to the orthogonal position. The artifact extent was higher in the 3 T MRI scanner compared to 1.5 T, this became more obvious in case of the titanium screw. Table 3, Figs. 2 and 3 summarize the results of the artifact size depending on screw position. Figs. 4 and 5 illustrate the artifacts in MDCT and FPCT.

**Table 3:** Artifact size of the screws implanted in chicken tibia in different screw positions (parallel (0°) and perpendicular (90°) to the longitudinal scanner axis) and different modalities

Imaging modality	Mg: mean artifact size [mm]	Ti: mean artifact size [mm]	Mean difference in artifact size [mm]	T-value	*P*-value
DX	0^a^	3.9 ± 0.3	3.9 ± 0.2	21.0	<0.001
MDCT					
0°	0^a^	14.1 ± 1.2	14.1 ± 1.2	45.5	<0.001
90°	0^a^	19.7 ± 1.4	19.7 ± 1.4	54.5	<0.001
FPCT					
0°	5.7 ± 0.3	14.1 ± 0.7	8.5 ± 0.2	45.1	<0.001
90°	6.9 ± 0.4	27.0 ± 2.4	20.1 ± 0.6	32.0	<0.001
1.5 T					
PDw TSE (FS) 0°	4.6 ± 0.5	6.2 ± 0.2	1.6 ± 0.1	12.0	<0.001
90°	7.8 ± 0.3	12.1 ± 0.1	4.3 ± 0.1	53.7	<0.001
1.5 T					
T1w TSE					
0°	3.5 ± 0.4	5.3 ± 0.2	1.8 ± 0.1	15.6	<0.001
90°	4.7 ± 0.2	9.0 ± 0.2	4.3 ± 0.1	58.9	<0.001
3 T					
PDw TSE (FS)					
0°	4.7 ± 0.4	8.6 ± 0.2	3.9 ± 0.1	33.8	<0.001
90°	8.4 ± 0.2	15.4 ± 0.3	7.0 ± 0.1	75.2	<0.001
3 T					
T1w TSE					
0°	4.5 ± 0.3	8.2 ± 0.2	3.7 ± 0.1	39.7	<0.001
90°	6.7 ± 0.2	10.3 ± 0.4	3.6 ± 0.1	31.2	<0.001
3 T					
PDw TSE WARP (FS)					
0°	4.7 ± 0.3	8.5 ± 0.2	3.8 ± 0.1	40.8	<0.001
90°	8.3 ± 0.2	13.4 ± 0.5	5.1 ± 0.1	36.7	<0.001

^a^In DX and MDCT the artifacts around the magnesium screw in chicken tibia were of such a small magnitude that they were innumerable and had to be considered zero

**Fig. 6:**
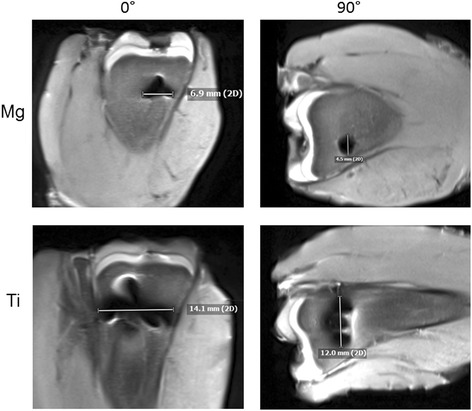
Artifact appearance in 3 T MRI PDw TSE (FS) in two positions of the screw (0°/90°)

Figure 6 shows the artifact appearance in MRI PDw TSE (FS). While the titanium screw generates significant signal distortion, the artifacts of the magnesium screw are almost completely restricted on the area of the implant.

**Fig. 7:**
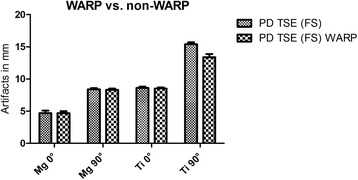
Magnesium and titanium screws in two positions (0°/90°) with a WARP application

Furthermore, in MRI scans a metal-artifact reduction sequence (WARP) was applied on both screws. When the scans were performed with the screws embedded in ultrasound gel, the artifacts of the titanium screw could be effectively reduced using WARP (mean reduction of 1.7 mm, t = 5.4, *p* < 0.001). For the magnesium screw, however, mean reduction was 0.36 mm and statistically not significant (*p* > 0.2). When implanted in chicken tibia, the WARP sequence showed for both screws a tendency towards artifact reduction regardless of screw position (as shown in Fig. 7), but without reaching the level of statistical significance.

The raw data of all measurements is displayed in Additional file 1.

## Discussion

In recent years magnesium alloys have been in the focus of research as degradable biomaterial. Several studies offered promising findings about magnesium implants and enabled the biomaterial to enter orthopedic routine.

Biomechanical data of a magnesium based screws by Waizy et al. suggested a promising bone-screw-fixation and therefore a great potential for medical application [10]. In a subsequent animal study with 15 rabbits he examined the screw with regard to degradation, bioactivity and biocompatibility [11]. While some parameters like clinics, radiography or blood samples could be obtained in vivo, for more precise analysis the animals had to be sacrificed. This provided the possibility to get histological results as well as to perform μCT scans in order to investigate the bone-implant interaction. It could be shown that new bone formed around the magnesium alloy implant indicating a good osteoinductivity [11]. Several other studies provided comparable results supporting the suitability of magnesium as a degradable biomaterial [12–20].

The main objective of this study was to evaluate metal artifacts of a magnesium based Herbert screw in different modalities applicable to patients in clinical routine. In some previous studies it is mentioned that magnesium implants do not interfere with common imaging modalities for post-op care, such as MRI and CT [4]. However, there is a lack of systematic studies with a direct comparison of different implants and modalities.

In 2005 Eggebrecht et al. could show an MRI compatibility of a coronary stent. One week after operation the stented coronary segment could be well visualized by MRI because of the absence of metallic artifacts [21]. In 2009 an animal cadaveric study by Ernstberger et al. verified that magnesium spinal implants produce significantly less artifacts in MRI than titanium spacers. Magnesium alloys behaved more like carbon fiber reinforced polymers with regard to the artifact areas [22, 23]. There is one previous study by Filli et al. in 2014 which systematically compared metal-induced artifacts in CT and MRI of a biodegradable magnesium alloy versus titanium and stainless steel controls. The result was that magnesium induces substantially fewer artifacts in CT and MRI in comparison to stainless steel. Compared to titanium artifacts on MRI were also less, but the results were statistically not significant. Only pins under standardized conditions in phantoms without surrounding tissue were tested [24], while we performed a cadaver study with a 3 T-MRI scanner, which is more relevant for musculoskeletal imaging in clinical routine. To the best of our knowledge, no study about artifacts induced by clinically applied biodegradable orthopedic implants has been published so far.

CT imaging is the gold standard for the evaluation of orthopedic hardware. It can be used to demonstrate the exact position of the material and its anatomic relationship with respect to adjacent structures. In the late postoperative period it is applied for the evaluation of bone fusion and detection of complications like loosening, migration or fracture of the implant [25–28]. However, artifacts generated by metallic implants can substantially degrade image quality. Imaging and reconstruction parameters as well as composition, shape and location of the hardware can significantly influence the extent of artifacts [25]. The results of our study are compatible with the known principles of artifact formation. The magnesium screw has a lower X-ray beam attenuation coefficient than titanium and therefore generates fewer artifacts. We could also show that the position of the screw is a crucial factor as the least artifacts arise when the X-ray beam passes the implant at its smallest diameter [25].

In MRI metal artifacts near metallic implants arise from local magnetic field inhomogeneities which are caused by large differences between the magnetic properties of human tissue and those of the implanted metal. The more substantial the difference in magnetic susceptibilities between the metallic object and the surrounding tissue is, the more severe are the artifacts [29–31]. Titanium implants are non-ferromagnetic and produce much less artifacts than ferromagnetic materials such as stainless steel. In our study we could show that compared to magnesium, the titanium screws showed significantly more signal loss and displacement artifacts such as geometric distortion and signal pile-up. This can be subscribed to the lower (mass) susceptibility of magnesium (6.9 × 10^−9^) compared to titanium (4.01 × 10^−8^) [1, 30].

In addition, we applied dedicated metal artifact reduction sequences (WARP TSE) and scanned the screws solely embedded in ultrasound gel. While artifacts of the titanium screw could be reduced using WARP, the mean reduction for artifacts of magnesium implants was too low and did not reach statistical significance. This can be explained by the fact, that the smaller the artifacts are, the more difficult it is to reduce them considerably and measurably. When implanted in chicken tibia, there was for both screws only a tendency towards artifact reduction without statistical significance. Due to the surrounding soft tissue it is even more difficult to achieve an accurate measurement of artifact differences smaller than 1 mm. In further studies it will be imported to apply more artifact reduction sequences, e.g. advanced WARP.

The major limitation of this study is that it was performed with a low number of specimens due to the lack of availability of implants. In order to acquire an adequate data volume for statistical analysis the scans were repeated at least five times after minimal repositioning of the specimens and the artifacts measurements were performed on at least three different slices.

Another limitation is the fact that the screws were scanned immediately after implantation in a cadaver. Therefore the degradation process which would normally occur in a patient was not analyzed. Further animal or even clinical studies are required to capture the influence of biodegradation on imaging.

Moreover, there is a limited standardization and reproducibility of the artifact measurement as there is no objective threshold for artifact extent. Also the orientation of the screws 0° and 90° to the z-axis of the scanner could not be achieved precisely and varied by a few degrees depending on the anatomy of the chicken cadaver. However the assessment of all artifacts was performed in an axial plane or reconstruction of the screws as they are major for the examination of the implant-bone interface. Furthermore there was a thin layer of air around the screw due to predrilling of the bone and implantation of the screw. Although this might reflect clinical reality, the high density contrast is very challenging for CT and might have influenced the artifact measurements. For future studies it is important to take the postoperative air into consideration as gas might be present both, after implantation as well as during the biodegradation process of Mg alloys. As the metallic artifacts of magnesium implants are significantly lower, the implant-bone interface can be depicted very precisely. Our results suggest, that there is a need to image magnesium implants after implantation with special regard to the precise distinction of air, gas formation and artifacts around the metal. It will be important to recognize the normal resorption process correctly and to differentiate from complications, e.g. implant loosening. Another objective for in vivo imaging of magnesium implants will be to detect soft tissue changes around the implant such as edema, inflammation, sclerosis. Imaging of the bone-implant interface is possible with both, CT and MRI, allowing for better understanding of these processes.

## Conclusions

In conclusion, magnesium Herbert screws show significantly fewer artifacts in DX, MDCT, FPCT and MRI in comparison to conventional titanium. Since biodegradable magnesium implants are commercially available in Europe and will now be increasingly used this knowledge is very important for postoperative imaging. Further in vivo studies are required in order to depict and understand the degradation and resorption process.

## Additional file

Additional file 1: Tables with the measured values for each modality, scan and screw. (PDF 448 kb)
