# Supplementary material for: Biodegradable magnesium Herbert screw – image quality and artifacts with radiography, CT and MRI
Source: BMC Med Imaging. 2017 Feb 14;17:16. doi: 10.1186/s12880-017-0187-7 (PMC5310087; doi:10.1186/s12880-017-0187-7)
Supplement: Additional file 1: — Tables with the measured values for each modality, scan and screw. (PDF 448 kb) [file 12880_2017_187_MOESM1_ESM.pdf]

Mg 0°

Scan number	MDCT	FPCT	MRI 1.5T (Avanto)		MRI 3T (Skyra)		
			PDw TSE (FS)	*T1w TSE (FS)*	*PDw TSE (FS)*	*T1w TSE (FS)*	*PDw TSE (FS)*
1	0	5.5	4.1	3.4	4.9	4.7	5.2
	0	5.5	4.2	3.3	4.9	4.6	5
	0	5.4	3.9	3.2	4.9	4.6	5.1
2	0	5.7	4.8	3.5	4.5	4	4.4
	0	5.8	4.8	3.4	4.6	3.9	4
	0	5.9	4.8	3.3	4.4	3.8	4.3
3	0	6	5.3	4.2	5.3	4.5	5
	0	6.1	4.8	4.1	5.2	4.4	5
	0	6	4.9	4	5.3	4.3	5.1
4	0	5.5	5	3	4.4	4.8	4.6
	0	5.6	5	3	4.1	4.8	4.5
	0	5.7	5.1	3.2	4.3	4.9	4.7
5	0	5.3	3.8	3.9	4.9	4.5	4.6
	0	5.4	4	3.8	4.7	4.5	4.7
	0	5.5	3.9	3.8	4.7	4.6	4.7
	0	5.66	4.56	3.54	4.74	4.46	4.72666667
	0	0.241661	0.49504209	0.38262253	0.35175749	0.32	0.32957886
Starie result	0	6.3			4.3	3.9	4.1

	Ti 0°				
	Scan number	MDCT	FPCT	MRI 1.5T (Avanto)	
WARP				***PDw TSE (FS)***	***T1w TSE (FS)***
	1	15.8	15.1	6.2	5.3
		15.7	15.1	6.2	5.3
		15.6	15.3	6.1	5.2
	2	13.9	14.1	6.4	5.2
		13.8	14.3	6.3	5.1
		13.9	13.9	6.3	5.2
	3	12.4	14.5	6.3	5
		12.6	14.6	6.3	5.1
		12.5	14.4	6.4	5.2
	4	13	13.2	5.9	5.5
		13.1	13.2	5.8	5.4
		13.2	13.3	6	5.6
	5	15.2	13.6	6.2	5.5
		15.4	13.5	6.1	5.7
		15.2	13.7	6.2	5.7
Mittlwert		14.08667	14.12	6.18	5.33333333
Std.Abweichung		1.230104	0.685274	0.168126936	0.21499354
		7.5	17.3		

MRI 3T (Skyra)			
PDw TSE (FS)	***T1w TSE (FS)***	***PDw TSE (FS) WARP***	
8.6	8.2	8.9	
8.6	8.1	8.8	
8.5	8	8.7	
8.9	7.9	8.6	
8.8	8	8.5	
8.7	8.1	8.3	
8.4	8	8.3	
8.3	8.1	8.2	
8.1	8.2	8.1	
8.6	8.1	8.5	
8.7	8.2	8.4	
8.8	8.3	8.3	
8.9	8.3	8.6	
8.8	8.4	8.6	
8.8	8.5	8.5	
8.63333333	8.16	8.486667	Mittlwert
0.22110832	0.15832456	0.215613	Std.Abweichung
7.5	5.9	5.6	
